# Sarcoidosis with cryptococcal infection apparently engaging only immune-privileged body compartments: a case report

**DOI:** 10.1186/s12879-020-05174-5

**Published:** 2020-06-22

**Authors:** Johannes H. van der Stoep, Eva Sigstad, Anders Bredberg

**Affiliations:** 1 Department of Pathology, Innlandet Hospital Trust, Lillehammer, Norway; 2 Department of Pathology, Oslo University Hospital, Oslo, Norway; 3 Medical Microbiology, Innlandet Hospital Trust, Lillehammer, Norway

**Keywords:** *Cryptococcus neoformans*, Sarcoidosis, Immunosuppression, Thyroid cancer

## Abstract

**Background:**

Infection with the *Cryptococcus neoformans* yeast fungus is largely restricted to patients with HIV, sarcoidosis or immunosuppressive therapies. In sarcoidosis, there is intense local immune response in granuloma lesions, coupled with a paradoxical systemic anergy. An analysis of cryptococcal infection in sarcoidosis may therefore shed light on whether opportunistic pathogens preferentially engage immune-privileged tissues.

**Case presentation:**

A 54-year-old man was admitted to our hospital after 2 months with palpitations and activity-related presyncope. A chest X-ray was normal, electrocardiography showed type-II atrioventricular-block, and there was a tentative diagnosis of myocarditis. Computed tomography reported minor hilar lymph glands and multiple nodular lesions in the lungs. Magnetic resonance imaging showed oedema and nodular structures in the heart, and fibrosis and granulomas were found in endomyocardial biopsies. The diagnosis was revised to cardiac sarcoidosis, and prednisone was initiated. In parallel, positron-emission tomography had revealed a marked uptake in the right thyroid lobe, a thyroid lobectomy was then performed, and the pathology showed a BRAF-positive papillary thyroid carcinoma. Four days postoperatively the patient developed symptoms suggestive of meningoencephalitis. Cerebrospinal fluid and blood cultures yielded growth of *C. neoformans*. Fungal staining of the thyroid specimen revealed cryptococcal elements in the carcinoma and in granulomas close to the tumour. Notably, there was no evidence of cryptococci in the heart sarcoid sections or in the normal thyroid parenchyma.

The patient was successfully treated with antifungal agents and at the 2-year follow-up there was no evidence of thyroid cancer relapse.

**Conclusion:**

This sarcoidosis patient had a remarkable clinic with evidence of cryptococcal infection only in body compartments commonly regarded to be immune-privileged. The findings suggest that an opportunistic and environmentally abundant pathogen, when infecting an immunocompromised host, primarily engages immunodeficient locations such as the brain, a tumour microenvironment and some forms of granuloma.

## Background

*Cryptococcus neoformans* is a yeast fungus that only rarely causes human disease although it is a common part of our environment due to soil contamination with bird excretions [1]. Its pathogenic role is largely limited to patients with HIV infection, sarcoidosis and immunosuppressive therapies [1, 2]. Sarcoidosis is an inflammatory disease with unknown etiology. A diagnostic hallmark is granulomas that can appear in virtually any organ, and most often in the lungs and hilar lymph glands [3]. While granuloma formation depends on CD4+ T cells and represents an intense local immune response, there is a paradoxical systemic anergy with loss of tuberculin reactivity and with cryptococcal infection reported in therapy-naïve patients [3, 4]. Here, we describe a sarcoidosis patient with a newly diagnosed thyroid carcinoma and with a *C. neoformans* infection apparently engaging only locations considered to be immune-privileged, as defined by a limited T cell and humoral immune response to antigens [5]. This case illustrates how an opportunistic pathogen can affect primarily immunosuppressed tissues within an immunosuppressed host.

## Case presentation

**Fig. 1:**
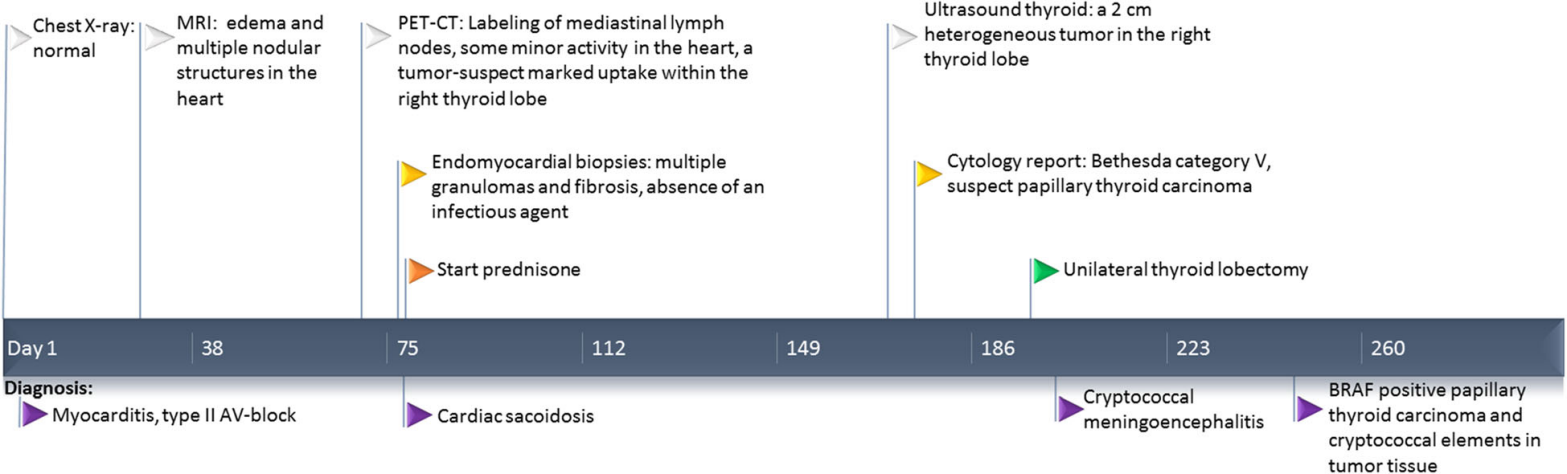
**A timeline clinical over-view.** The violet flags below the timeline indicate the consecutive diagnoses. White flag, imaging analyses; yellow flags, biopsy and cytology; orange flag, immunosuppressive therapy; green flag, surgery

Against a background of hypertension and migraine, a 54-year-old man was admitted to our hospital after 2 months with palpitations and activity-related presyncope, as well as influenza-like symptoms. Chest X-ray was normal. Electrocardiography showed type-II atrioventricular-block. A tentative diagnosis of myocarditis was made. Figure 1 gives a timeline presentation of the successive diagnoses and the main clinical investigations.

**Fig. 2:**
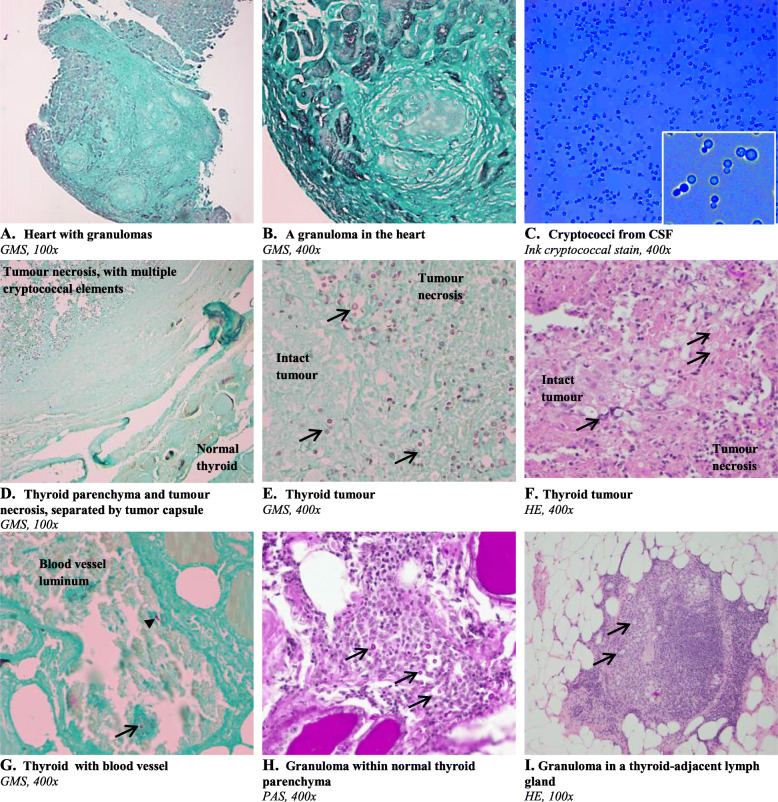
**Histological sections and cerebrospinal fluid microscopy.** Arrows indicate characteristic cryptococcal elements with the capsule appearing as an unstained clear halo. Endomyocardium with granulomas but with no evidence of cryptococci **a** and **b**, CSF finding of cryptococci **c**, thyroid tumour necrotic area with cryptococci but with no signs of cryptococci in the adjacent normal thyroid glandular tissue **d**, thyroid tumour necrosis and focal intact tumour tissue **e**, thyroid tumour with cryptococci and inflammatory infiltrate **f**, thyroid parenchyma without cryptococci except for within a blood vessel, note budding indicated by the arrowhead **g**, cryptococcal elements in epithelioid cell granulomas in the thyroid parenchyma **h**, cryptococcal elements in granulomas in a thyroid-adjacent lymph gland **i**. Staining types are cryptococcal ink, hematoxylin and eosin (HE), the fungal-specific Grocott’s methenamine silver (GMS) stain and Periodic acid–Schiff (PAS)

Three weeks later, magnetic resonance imaging (MRI) showed oedema and multiple nodular structures in the heart. Positron-emission tomography (PET) revealed uptake by mediastinal lymph glands, some minor activity in the heart, and focal marked uptake in the right thyroid lobe, suspicious of malignancy. Chest computed tomography (CT) reported minor (10 mm) hilar lymph glands and multiple 2–5 mm nodular lesions in the lungs. Endomyocardial biopsies showed fibrosis and granulomas, in the absence of infectious agents (Figs. 2a, b), prompting a diagnosis of cardiac sarcoidosis. An automated defibrillator was implanted and corticosteroid therapy (prednisone) initiated.

Three months later, a fine needle aspirate of the thyroid was reported as suspicious for papillary thyroid carcinoma (PTC). A thyroid lobectomy was performed 27 days later, and pathology showed a BRAF-positive encapsulated PTC measuring 28 mm with diffuse necrosis. Four days postoperatively the patient developed signs and symptoms of meningoencephalitis. There were 39 leukocytes per μl cerebrospinal fluid (CSF) (with both polynuclear and mononuclear cells). Blood samples revealed the same findings as during the preceding 3 months; granulocytosis and minor lymphopenia. Antiviral therapy was started, but CSF and blood cultures 2 days later yielded growth of *C. neoformans* (Fig. 2c), identified at species level using matrix-assisted laser desorption/ionization-time-of-flight mass spectrometry (MALDI-TOF MS).

Fluconazole (800 mg) was given intravenously before the patient was transferred to a central hospital and the antifungal regimen was then changed to amphotericin B (a daily 200 mg infusion) plus flucytosine (1500 mg infusion twice daily); one month later parenteral treatment was terminated and flucytosine (1500 mg × 2) plus fluconazole (400–800 mg daily) were given during the next month, to be followed by fluconazole (400 mg) until 1 year. Cerebrospinal fluid was sampled weekly, showing sparse growth of *C. neoformans* after 1 week while later samples were without growth but with decreasing quantity documented by a *C. neoformans* antigen test until 2 months later. Neurological status including cognitive function was noted to be fully normalized at 1 month after onset of the cryptococcal infection. There were no signs of cryptococcus infection at follow-up 15 months after diagnosis, and antifungal treatment was terminated.

A retrospective fungal staining of the thyroid specimen revealed widespread cryptococcal elements in the PTC (Figs. 2d-f). In addition, cryptococcal elements were observed intravascularly (Fig. 2g), in the few non-necrotizing granulomas located in the thyroid parenchyma (Fig. 2h) as well as in an adjacent lymph gland (Fig. 2i). The types and distribution of cells within the thyroid granulomas (Fig. 2h) were typical for those commonly seen upon histopathologic examination of granulomatous inflammation, with centrally located giant cells and epitheloid histiocytes dominating, and with lymphocytes only at the outer rim [6]. Notably, there was no evidence of cryptococci in the heart sarcoid sections (Figs. 2a, b) or in non-cancerous thyroid parenchyma adjacent to areas with tumour transformation (Figs. 2d, g, h).

At the 2-year follow-up there was no evidence of thyroid cancer relapse, but the cardiac sarcoidosis was still active.

## Discussion and conclusions

The immune hyper-activity observed in sarcoidosis is thought to be limited to affected organs, being in line with our observation that the cryptococcal infection did not involve the heart including granulomas presumed to be of sarcoid type. Conversely, a paradoxical systemic anergy in sarcoidosis has been documented, with enrichment of regulatory T cells in peripheral blood and loss of tuberculin reactivity [3] being in line with our finding of cryptococci only at three immune-privileged locations [5, 7–9].

Our finding of a normal thyroid parenchyma with no signs of infection, together with cryptococcal elements in adjacent thyroid cancer and granuloma section areas is intriguing. However, this is in line with previous reports showing that cryptococcal infection engaging the thyroid gland is rare, and only sporadically documented in HIV-negative subjects, as reviewed by Avram et al. [10]. A retrospective autopsy study on HIV-positive cases reported that cryptococci were found in the thyroid in no more than one out of the total of 102 individuals [11].

It may be speculated that the cryptococcal infection contributed to the development of cardiac sarcoidosis. Cryptococcal meningitis is thought to be preceded by inhalation of the fungus and an up to year-long phase of a subclinical lung infection [1]. Sarcoidosis is assumed to be elicited in predisposed individuals by one among an array of etiological agents including low-virulent pathogens [4]. Therefore, the following timeline of events in our case is possible: a subclinical pulmonary cryptococcal infection passes into a resolution (or spreading) phase with anti-cryptococcal immune activity eliciting the sarcoidosis; immunosuppressive therapy is initiated; the cryptococci will be able to disseminate and survive at immune-privileged sites, and cause meningitis. The source of the disseminated cryptococcal infection phase may also include the thyroid tumor and granulomas observed by us to harbour this fungus already before the onset of this advanced infection phase.

Our patient had a remarkable and informative clinic with cryptococcal infection documented by us only in body compartments commonly regarded to be immune-privileged; the brain [5], a malignant tumour [8, 9] and chronic granulomas [7]. In conclusion, this case report suggests that an opportunistic and environmentally abundant pathogen, when infecting an immunocompromised patient, primarily engages immune-privileged locations such as the brain, tumour microenvironment and some forms of granuloma.

## Data Availability

All the information supporting our conclusions are included in the manuscript. There are no datasets related to this case report.

## Abbreviations

CSF: Cerebrospinal fluid
CT: Chest computed tomography;
GMS: Grocott’s methenamine silver
HE: hematoxylin and eosin
MRI: magnetic resonance imaging
PAS: Periodic acid–Schiff
PET: Positron-emission tomography
PTC: Papillary thyroid carcinoma
